# Supplementary material for: Clade IIb Mpox virus (MPXV) vertical transmission and fetal demise in a pregnant rhesus macaque model
Source: PLoS One. 2025 Apr 1;20(4):e0320671. doi: 10.1371/journal.pone.0320671 (PMC11960918; doi:10.1371/journal.pone.0320671)
Supplement: S1 Fig — (DOCX) [file pone.0320671.s001.docx]

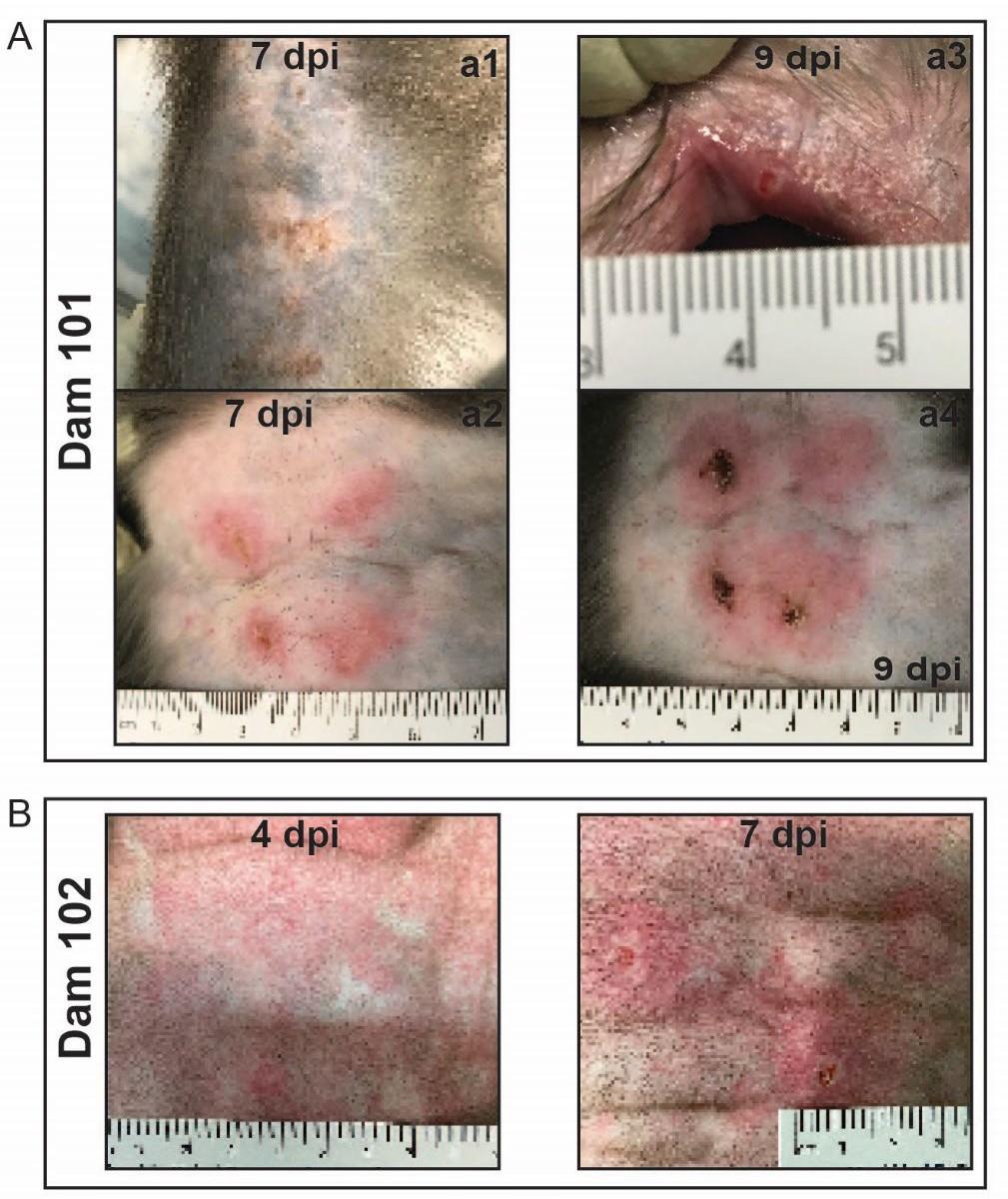


**Supplemental Figure 1. Skin lesion appearance.** (**A**) Skin lesions first appeared on dam 101 at 7 days post- inoculation (dpi). (**a1**) Multiple pustules with superficial serocellular crusting appeared on an arm distant from the inoculation sites. (**a2**) Large pustules and dermal ulcerations surrounded by erythema appeared at the sites of inoculation between the scapula with smaller macules, papules, and pustules developing adjacent and between inoculation sites. (**a3**) By 9 dpi, there was focal ulceration of the oral mucocutaneous junction. (**a4**) By 9 dpi, injection site ulcers had developed eschars and were still surrounded by intense erythema as well as small pustules and ulcers. (**B**) Skin lesions first appeared on dam 102 at 4 dpi. The skin inoculation sites between the scapulae were erythematous macules and papules. By 7 dpi, the inoculation sites progressed to ulcers and a papule surrounded by erythema
