# Supplementary material for: Clade IIb Mpox virus (MPXV) vertical transmission and fetal demise in a pregnant rhesus macaque model
Source: PLoS One. 2025 Apr 1;20(4):e0320671. doi: 10.1371/journal.pone.0320671 (PMC11960918; doi:10.1371/journal.pone.0320671)
Supplement: S2 Fig — (DOCX) [file pone.0320671.s002.docx]

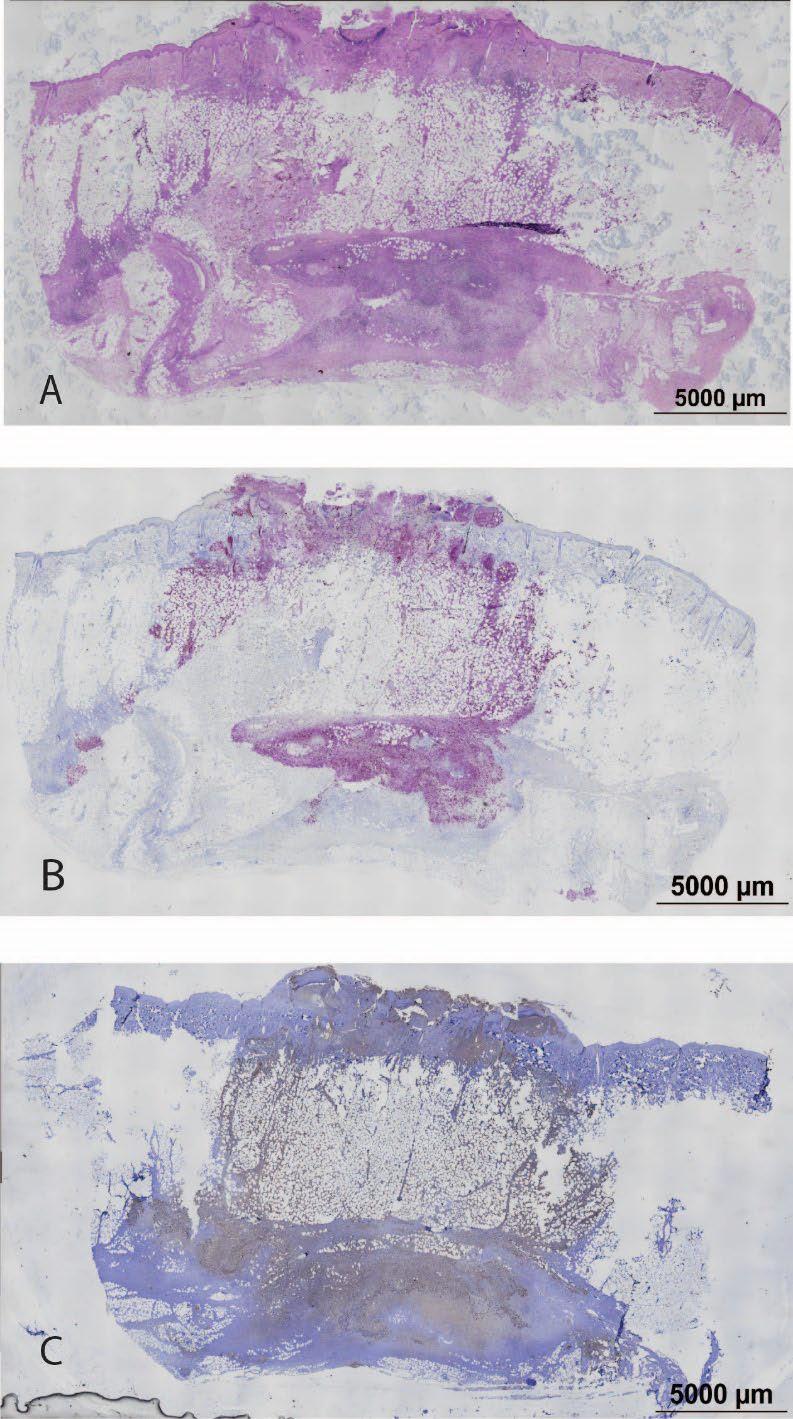


**Supplemental Figure 2. Maternal skin lesion histology and MPXV localization by ISH and IHC.** (**A**) Hematoxylin and eosin staining of 5µM section. There is focally extensive epidermal and dermal ulceration, necrotizing dermatitis, steatitis of the underlying subcutaneous adipose, and severe diffuse neutrophilic, lymphoplasmyctic and occasionally eosinophilic and histiocytic panniculitis with multifocal vasculitis and vascular necrosis. The intact skin on either side of the ulcer has multiple epidermal pustules, ballooning degeneration of keratinocytes, and moderate to severe multifocal dermatitis. (**B**) ISH signal (red) and hematoxylin (blue) staining within the ulcer, dermis, subcutis, and panniculus of a serial skin section. (**C**) IHC signal (brown) and hematoxylin (blue) staining within the ulcer, dermis, subcutis, and panniculus of a nearby skin section.
