# Supplementary material for: Clade IIb Mpox virus (MPXV) vertical transmission and fetal demise in a pregnant rhesus macaque model
Source: PLoS One. 2025 Apr 1;20(4):e0320671. doi: 10.1371/journal.pone.0320671 (PMC11960918; doi:10.1371/journal.pone.0320671)
Supplement: S3 Fig — (DOCX) [file pone.0320671.s003.docx]

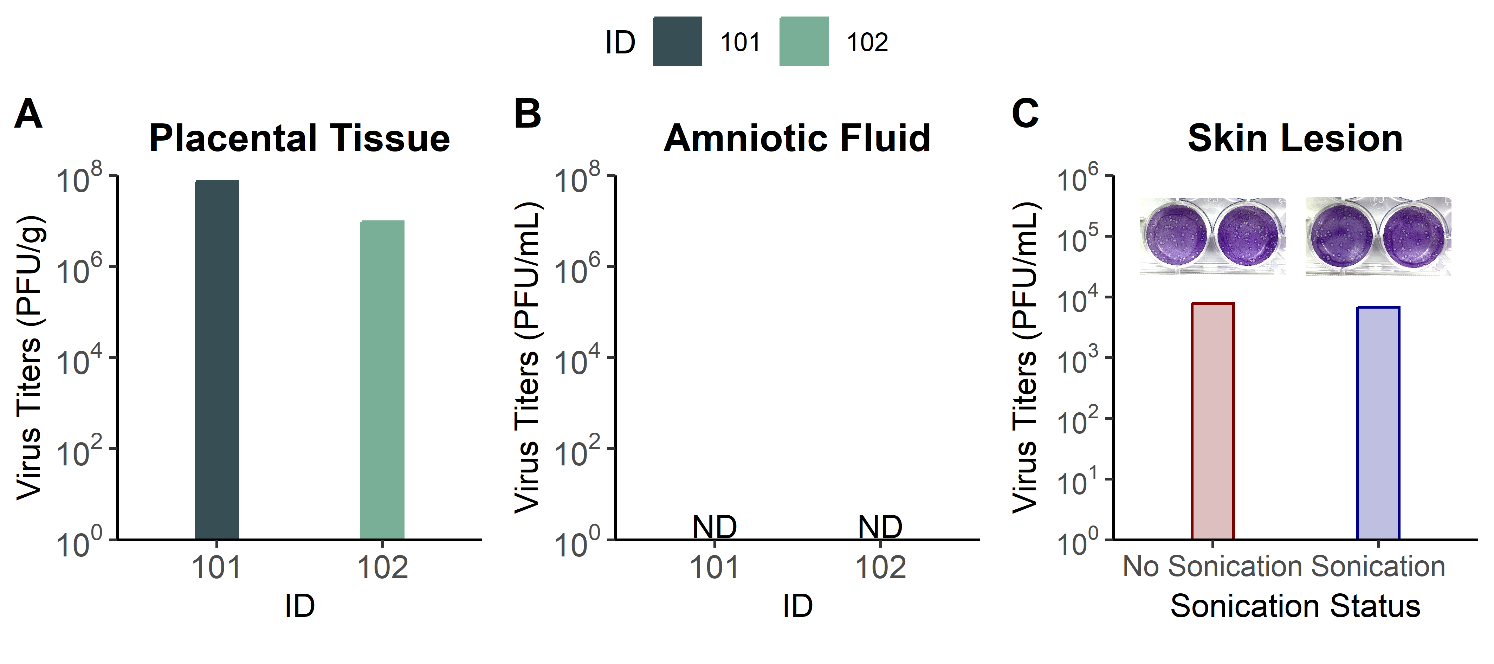


**Supplemental Figure 3. Placental tissue and amniotic fluid infectious virus titers and sonication comparison.** MPXV infectious virus titers were measured via plaque assay at the time of necropsy in placental tissue homogenates (**A**) and amniotic fluid (**B**) for IDs 101 and 102. For ID 103, no plaque assays were performed because viral DNA titers were below the limit of detection. The impact of performing sonication on infectious virus titers was determined using an eluate from a skin lesion swab from ID 101 at 7 days post-inoculation (**C**). Representative images show plaque formation following a 1:10 dilution of sample that was either not sonicated or sonicated.
