# Supplementary material for: Clade IIb Mpox virus (MPXV) vertical transmission and fetal demise in a pregnant rhesus macaque model
Source: PLoS One. 2025 Apr 1;20(4):e0320671. doi: 10.1371/journal.pone.0320671 (PMC11960918; doi:10.1371/journal.pone.0320671)
Supplement: S5 Fig — (DOCX) [file pone.0320671.s005.docx]

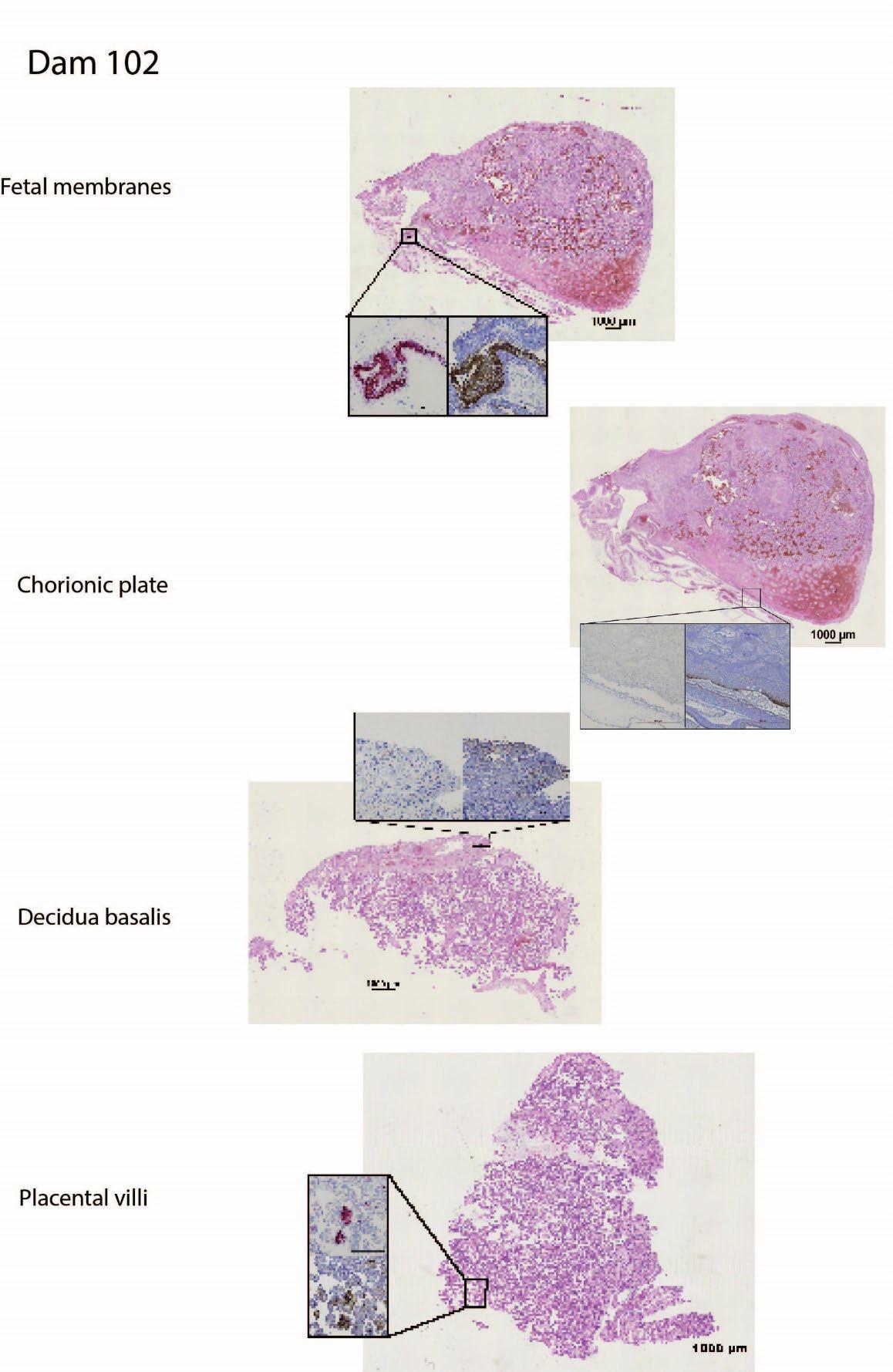


**Supplemental Figure 5. Full thickness sections shown in figure 3 for dam 102 demonstrating the location where each insert was located in the full thickness section.**
