# Supplementary material for: Clade IIb Mpox virus (MPXV) vertical transmission and fetal demise in a pregnant rhesus macaque model
Source: PLoS One. 2025 Apr 1;20(4):e0320671. doi: 10.1371/journal.pone.0320671 (PMC11960918; doi:10.1371/journal.pone.0320671)
Supplement: S6 Fig — (DOCX) [file pone.0320671.s006.docx]

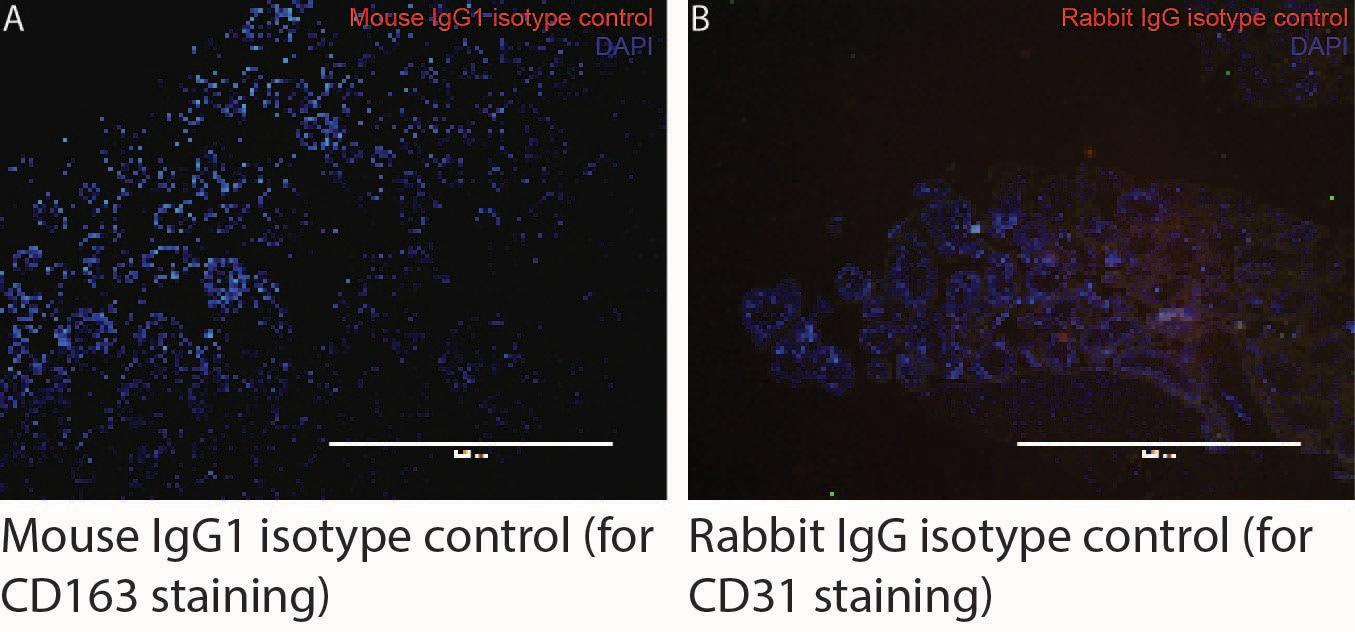


**Supplemental Figure 6. Isotype control staining for immunofluorescence imaging.** (**A**) Placental tissue section stained with DAPI and mouse IgG1 antibody as an isotype control for the CD163 mouse IgG1 antibody. (**B**) Placental tissue section stained with DAPI and rabbit IgG antibody as an isotype control for the CD31 rabbit IgG antibody.
