# Supplementary material for: Clade IIb Mpox virus (MPXV) vertical transmission and fetal demise in a pregnant rhesus macaque model
Source: PLoS One. 2025 Apr 1;20(4):e0320671. doi: 10.1371/journal.pone.0320671 (PMC11960918; doi:10.1371/journal.pone.0320671)
Supplement: S1 Table — (DOCX) [file pone.0320671.s007.docx]

| **Reagent** | **Manufacturer** | **Product number** | **Dilution** | **Assays in which reagent was used** |
| --- | --- | --- | --- | --- |
| Rabbit anti Vaccinia | Genetex | GTX36578 | 1:1000 (IHC)  or 1:1500 (IF) | IHC primary, IF primary |
| Mach 2  Rabbit HRP-  polymer | Biocare | RHRP520H | Neat | IHC secondary |
| CD163 | Genetex | GTX42365 | 1:100 | IF primary |
| Cytokeratin | Sigma | 452M-94 | 1:75 | IF primary |
| CD31 | Bioss | BSM10825M | 1:200 | IF primary |
| Goat anti rabbit IgG (H+L) Alexa fluor 647 | Invitrogen | a32728 | 1:1000 | IF secondary |
| Goat anti rabbit IgG (H+L) Alexa fluor 594 | Invitrogen | a32740 | 1:1000 | IF secondary |
| Rabbit IgG  isotype control | Invitrogen | 31236 | 1:4000 | Control = IHC for vaccinia and IF for vaccinia |
| Mouse IgG2 | Santa Cruz | 3878 | 1:100 | Control = IF for cytokeratin |
| Mouse IgG1 | Santa Cruz | 3877 | 1:100 | Control = IF for  CD163 and CD31 |

**Supplemental Table 1. Table of reagents used in immunohistochemistry (IHC) and immunofluorescence (IF) experiments.**
