# Supplementary material for: Clade IIb Mpox virus (MPXV) vertical transmission and fetal demise in a pregnant rhesus macaque model
Source: PLoS One. 2025 Apr 1;20(4):e0320671. doi: 10.1371/journal.pone.0320671 (PMC11960918; doi:10.1371/journal.pone.0320671)
Supplement: S2 Table — (DOCX) [file pone.0320671.s008.docx]

| **Supplemental Table 2. MPXV stock sequence and variants.** | | | | | | | |
| --- | --- | --- | --- | --- | --- | --- | --- |
| **Single nucleotide polymorphisms detected at ≥25% frequency** | | | | | | |  |
| Position - ON563414.3 |  | Amino acid change | CDS position | Nucleotide change | Variant frequency | Genome location in relation to ON563414.3 |  |
| 34,325 |  | S -> L | 383 | G -> A | 58.2% | MPXVgp041 |  |
| 55,125 |  | A -> T | 34 | C -> T | 26.0% | MPXVgp059 |  |
| 119,451 |  | L -> F | 154 | G -> A | 62.4% | MPXVgp121 |  |
|  |  |  |  |  |  |  |  |
| **Deletions detected at ≥25% frequency** | | | | | | |  |
| Position - ON563414.3 | Length | Change | Coverage | Polymorphism Type | Variant Frequency | Genome location/protein product |  |
| 6 | 1 | -G | 402 | Deletion | 49.3% | inverted terminal repeat |  |
| 9 | 1 | -C | 406 | Deletion | 48.3% | inverted terminal repeat |  |
| 16 | 1 | -A | 423 | Deletion | 47.5% | inverted terminal repeat |  |
| 20 | 1 | -T | 423 | Deletion | 49.6% | inverted terminal repeat |  |
| 197,186 | 1 | -A | 474 | Deletion | 46.6% | inverted terminal repeat |  |
| 197,190 | 1 | -T | 474 | Deletion | 46.8% | inverted terminal repeat |  |
| 197,197 | 1 | -G | 461 | Deletion | 47.1% | inverted terminal repeat |  |
| 197,200 | 1 | -C | 457 | Deletion | 47.9% | inverted terminal repeat |  |
|  |  |  |  |  |  |  |  |
| **Insertions detected at >25% freqency and over 100bp in length** | | | | | | |  |
| Position - ON563414.3 | Length | Change | Coverage | Polymorphism Type | Variant Frequency | Genome location/protein product |  |
| 4,697 | 128 | (AACTAACTTATGACTT)8 -> (AACTAACTTATGACTT)16 | 381 | Insertion (tandem repeat) | 90.0% * | between coding sequences | * remaining 10% of reads contain between 11-19 repeat units |
| 179,074 | 342 | (CATTATATA)16 -> (CATTATATA)54 | 344 | Insertion (tandem repeat) | 84.6% * | between coding sequences | * remaining 15.4% of reads contain between 41-69 repeat units |
| 192,383 | 128 | (AGTCATAAGTTAGTTA)8 -> (AGTCATAAGTTAGTTA)16 | 412 | Insertion (tandem repeat) | 92.5% * | between coding sequences | * remaining 7.5% of reads contain between 11-19 repeat units |
|  |  |  |  |  |  |  |  |
| **Homopolymer/tandem repeat variants detected at >25% freqency** | | | | | | |  |
| Position - ON563414.3 |  | Change | Coverage | Polymorphism Type | Variant Frequency | Genome location/protein product |  |
| 613 |  | (T)14 -> (T)4-16 [most frequent variant: (T)12] | 461 | Insertion/deletion (homopolymer) | 83.1% | inverted terminal repeat |  |
| 133,095 |  | (T)28 -> (T)1-33 [most frequent variant: (T)17] | 320 | Insertion/deletion (homopolymer) | 97.2% | between coding sequences |  |
| 172,072 |  | (T)9 -> (T)3-12 [most frequent variant: (T)8] | 401 | Insertion/deletion (homopolymer) | 36.4% | between coding sequences |  |
| 173,267 |  | (AT)24 -> (AT)10-30 [most frequent variant: (AT)25] | 391 | Insertion/deletion (tandem repeat) | 75.2% | between coding sequences |  |
| 196,580 |  | (A)14 -> (A)1-17 [most frequent variant: (A)13] | 504 | Insertion/deletion (homopolymer) | 83.5% | inverted terminal repeat |  |
